# The Relationship Between Spiritual Intelligence and Compliance with Professional Values in Nursing Students in Türkiye

**DOI:** 10.1007/s10943-025-02291-w

**Published:** 2025-03-13

**Authors:** Sevda Korkut, Büşra Çetin

**Affiliations:** 1 Department of Nursing, Faculty of Health Sciences, Erciyes University, Kayseri, Türkiye; 2 Department of Nursing, Institute of Health Sciences, Erciyes University, Kayseri, Türkiye

**Keywords:** Nursing, Nursing students, Professional values, Spiritual intelligence

## Abstract

This study was conducted to reveal the relationship between nursing students’ spiritual intelligence levels and their compliance with professional values. This research was designed as a cross-sectional and correlational study. The study included 311 nursing students from a university in Türkiye. Descriptive characteristics form, spiritual intelligence scale and nurses professional values scale—revised were used to collect the research data. There was a moderate positive correlation between students’ nursing professional values and spiritual intelligence scores. Spiritual intelligence explained 10% of the total variance in compliance with professional values.

## Introduction

Spiritual intelligence is an internal capacity that gives an individual the ability to distinguish right from wrong, increase their flexibility when faced with difficulties and different situations and enable them to achieve self-awareness (Seyfi & Köse, 2016; Sharifnia, 2022; Zohar & Marshall, 2000). In other words, spiritual intelligence is the ability to utilize and embody spiritual qualities and resources to improve daily functioning and well-being (Amram, 2022). At the same time, spiritual intelligence is seen as a unifying type of intelligence that brings together cognitive, emotional and intellectual intelligence and is therefore considered to be the most superior form of intelligence (Amram, 2022; Skrzypinska, 2021).

Cognitive, social and emotional intelligence have been discussed as necessary elements for participation in professional practices and spiritual intelligence, which combines these elements, has made a name for itself in many professional groups in recent years. Recently, spiritual intelligence has been accepted as one of the basic skills needed by professionals and has rapidly become widespread in nursing practice (Amram, 2022; Beni et al., 2019; Sharifnia et al., 2022; Skrzypinska, 2021). Because nursing does not only require technical knowledge and skills, it is also a professional profession that involves responding to the physical, emotional and spiritual needs of patients. Additionally, nursing is one of the professions in which the role of multiple intelligences, including spiritual intelligence, is emphasized more than other health professions. Therefore, nursing utilizes spiritual intelligence to make sense of the individual and provide holistic care (Beni et al., 2019; Durmuş et al., 2018).

Spiritual intelligence enables nurses to create their goals throughout their lives, make sense of all their physical and mental experiences, and make personal inferences that will achieve their professional goals (Beni et al., 2019; Durmuş et al., 2018). In this case, spiritual intelligence appears as a factor affecting care (Sharifnia et al., 2022). Nurses with high spiritual intelligence have important consciousness about why they are in this profession, their position, and how they should use it (Beni et al., 2019; Sharifnia et al., 2022).

Spiritual intelligence is important for nurses who provide holistic and quality care. This situation is also necessary for nursing students who will practice nursing in the future. However, as the spiritual intelligence level of each individual is not the same, the spiritual intelligence level of nursing students is not the same (Ahmadi et al., 2021). In a study conducted on nursing students, the spiritual intelligence levels of students were measured and it was stated that many factors such as academic achievement, family attitude, social values and economic status were among the factors affecting spiritual intelligence. In addition, it was stated in the study that nurses with high levels of spiritual intelligence will increase the quality of care and this will directly positively affect the society. This situation leads to the belief that spiritual intelligence is a factor affecting nursing care (Durmuş et al., 2018).

Spiritual intelligence covers many dimensions, including general thinking, coping with problems, self-awareness and dealing with moral issues. While facilitating nurses to cope with complex and stressful clinical situations, it also ensures that the profession adheres to professional values by guiding ethical decision-making processes (Sharifnia et al., 2022). Professional values in nursing have been influenced by both technological developments and scientific research from past to present and have revealed their own values and have been important in making nursing a professional profession (Subaşı & Özbek Güven, 2022).

Professional values have the characteristics of being open to today’s technological developments, appropriate to evidence-based care, integrating with the community, compatible with the ethical values of nursing and respectful to human rights. These values begin to be learned through nursing education (Altun, 2021). Developing students’ spiritual intelligence in nursing education strengthens their ability to make ethical and value-based decisions in their future professional lives (Sharifnia et al., 2022). Because spiritual intelligence goes beyond the traditional approach and expands one’s awareness and perspective toward one’s profession (Pinto et al., 2023). Mehralian et al. (2023) found that nurses with high spiritual intelligence developed a stronger ethical sensitivity, fulfilled their professional responsibilities better and were more sensitive in patient care.

Professional values and spiritual intelligence levels of nursing students have an important place in terms of quality of care. When the literature is examined, there are studies on the role of professional values and spiritual intelligence in nursing care (Beni et al., 2019; Durmuş et al., 2018; Mehralian et al., 2023). However, there is no study examining the relationship between spiritual intelligence and professional values in nursing students. It is thought that examining the relationship between spiritual intelligence and compliance with professional values in nursing students will provide important information in terms of factors affecting the development of professional values in nursing education. In this context, this study was conducted to reveal the relationship between nursing students’ spiritual intelligence levels and their compliance with professional values.

### Hypothesis

The spiritual intelligence level of nursing students significantly effects their compliance with nursing professional values.

## Method

### Design

This research was conducted as a cross-sectional and correlational study.

### Participants and Setting

The study was conducted at the Nursing Department of the Health Sciences Faculty of a university in Türkiye. Nursing education in this nursing department consists of a total of eight semesters, two semesters each year. Students, who learn only theoretical courses and skill laboratory practices in the 1st year, begin to practice in clinical settings from the 2nd year. In the 4th year, students continue their integrated clinical/field practice throughout the semester. It is expected that students’ professional nursing values will also develop during the education process. In the 2023–2024 academic year, the department had a total of 970 students, 220 students each in the first, second and third grades and 310 students in the fourth grade.

In order to determine the research sample, it was calculated with the G*Power 3.1.9.4 program, taking into account the mean score of the “Professional Values Scale” in the study conducted by Çulha and Acaroğlu (2019). Accordingly, it was calculated that at least 266 students should be included in the sample with 5% sampling error, 85% power and tolerance rate not exceeding 0.05. Data were collected between May and July 2024. As a result of students' intense interest in the study, 311 students participated in the study. Then, a post-hoc power analysis was conducted to calculate the power of the research. In this analysis, the correlation coefficient between spiritual intelligence and professional values was taken as a reference. According to this calculation, in the bivariate normal model, *n* = 311, alpha = 0.05, 0.356 for “correlation ρ H1” and 0 for “correlation ρ H0”, the power of the research was found 99%.

The inclusion criteria are as follows: (1) speaking and understanding Turkish, (2) not having communication problems and (3) volunteering to participate in the study. Exclusion criteria were unwillingness to participate in the study. Trainings for the development of professional values of nursing students start to be given from the first grade. For this reason, no grade restriction was made in the study and all grades were included in the study.

### Data Collection

The convenience sampling method was used to reach the sample. The data collection process was carried out online via Google Forms. Normally, each class has a WhatsApp group to share announcements and information. The purpose of the study was explained to the students from these groups. Then, the link to the study was shared and students who met the inclusion criteria were asked to fill out the forms. It took the students an average of 15 min to complete these forms. In addition, no data containing student identification information or school numbers were collected in the forms. IP address limitation was also made through Google Forms so that each student could fill out the form only once. In addition, system warnings were created for the compulsory questions to be answered in the questionnaire forms and missing data were prevented.

### Data Collection Tools

Descriptive characteristics form, spiritual intelligence scale and nurses professional values scale—revised were used to collect the research data.

*Descriptive Characteristics Form* was prepared by the researchers. In this form, questions were asked about the demographic information of the students, their level of education on spirituality and their willingness to choose the nursing profession.

*Spiritual Intelligence Scale* is used to assess spiritual intelligence. The Turkish validation of the scale developed by Kumar and Mehta (2011) was conducted by Erduran-Tekin and Ekşi (2019). The scale consists of 19 items and four sub-dimensions. Its sub-dimensions are self-understanding, human values, compassion and conscience. The scale items are in 5-point Likert type. There are nine reverse-scored items in the scale (items: 1, 4, 7, 9, 13, 14, 15, 16, 17). The lowest score that can be obtained from the scale is 19 and the highest score is 95. The higher the score on the scale, the higher the spiritual intelligence. In the validity and reliability study, it was calculated that the goodness of fit statistics of the scale was at appropriate values, Cronbach’s Alpha reliability coefficient was 0.86 and 0.85, and it was stated that the scale was a valid and reliable measurement tool and could be used in future studies. In this study, the Cronbach’s Alpha reliability coefficient of the scale was found to be 0.708. In the correlation analysis used to test the relationship between spiritual intelligence and compliance with professional values, which is the main purpose of this study, the spiritual intelligence scale sub-dimension and total scale score were analyzed separately, taking into account the risk of contamination emphasized by Koenig and Carey (2024) (Koenig & Carey, 2024).

*Nurses Professional Values Scale—Revised* reflecting the American Nurses Association’s code of ethics was developed by Darlene Weis and Mary Jane Schank in 2009 (Weis & Schank, 2009). It was developed to determine the compliance of nurses and nursing students with professional values. The Turkish validity and reliability study of the scale was conducted by Acaroğlu (2014). The 5-point Likert-type scale consists of 26 items. Each item is scored between 5 (most important) and 1 (not important), and these scores are summed to obtain scale scores. Scores that can be obtained from the scale range between 26 and 130. A high score indicates a strong adaptation to professional values. The scale does not include sub-dimensions. In the scale independent of the sub-dimension, there are sub-factors named as caring, professionalism and trust that contribute to the interpretation of the data. The goodness of fit statistics of the scale was found to be at appropriate values, Cronbach’s Alpha reliability coefficient was calculated to be 0.96 and it was stated that the scale was a valid and reliable measurement tool and could be used in future studies. In this study, Cronbach’s Alpha coefficient of the total scale was 0.981.

### Data Analysis

Statistical analyses were performed using IBM SPSS Statistics 25.0 (IBM Corp, Armonk, NY, USA). Descriptive characteristics of the students are presented as number (n), percentage (%) and mean ± standard deviation (Mean ± SD) values. Shapiro–Wilk test and Q–Q plots were used for normality test. Pearson’s correlation coefficient was used to test the correlation between continuous variables and scales. Regression analysis was used to determine the predictive effect of spiritual intelligence, gender, choosing the profession willingly and receiving training on spirituality on professional values. In all analyzes, *p* < 0.05 was considered significant.

### Ethical Considerations

Compliance with ethical principles was emphasized at every stage of the study. Ethics committee approval (2024/111) was obtained from the University Ethics Committee and institutional permission was obtained from the institution where the research was conducted. The purpose of the study was firstly explained to the students who participated in the study. Then, the students who would participate in the study were allowed to continue the study by checking the box “I agree to participate in the study”. For the nurses professional values scale—revised used in the study, permission was obtained by e-mail from the author who conducted the adaptation study. For the spiritual intelligence scale, the scale was used in the study in accordance with the authors’ statements that “it can be used without permission with suitable citation”.

## Results

The mean age of the students participating in the study was 20.88 ± 2.61 and 85.9% were female. Of the students, 28.6% are studying in the first grade. While 59.2% of the students stated that they chose the nursing profession willingly, 63.3% stated that they had not received any education about spirituality before (Table 1).

**Table 1:** Descriptive characteristics of the students (*N* = 311)

Characteristics	*n* (%)
Age (Mean ± SD)	20.88 ± 2.61
*Gender*	
Female	267(85.9)
Male	44(14.1)
*Class*	
1st grade	89(28.6)
2nd grade	86(27.6)
3rd grade	72(23.2)
4th grade	64(20.6)
*Choosing the nursing profession willingly*	
Yes	184(59.2)
No	127(40.8)
*Receive training on spirituality*	
Yes	114(36.7)
No	197(63.3)

The mean score of the spiritual intelligence scale was 65.07 ± 7.47, and the mean scores of the self-understanding sub-dimension were 24.22 ± 3.41, human values 12.76 ± 2.78, compassion 16.49 ± 3.50 and conscience 11.59 ± 2.39. The mean score of the professional values scale was 98.87 ± 20.99, and the mean score of caring was 58.02 ± 12.51, professionalism was 29.83 ± 6.61 and trust factor was 11.00 ± 2.59 (Table 2).

**Table 2:** Scores of the spiritual intelligence scale and nurses professional values scale—revised

Scale	Mean ± SD	Min–Max	Scale Min–Max
*Spiritual intelligence scale*			
Self understanding	24.22 ± 3.41	14–35	7–35
Human values	12.76 ± 2.78	6–20	4–20
Compassion	16.49 ± 3.50	5–25	5–25
Conscience	11.59 ± 2.39	3–15	3–15
Overall scale	65.07 ± 7.47	42–92	19–95
*Nurses professional values scale—revised*			
Caring	58.02 ± 12.51	15–75	15–75
Professionalism	29.83 ± 6.61	8–40	8–40
Trust	11.00 ± 2.59	3–15	3–15
Overall scale	98.87 ± 20.99	26–130	26–130

There was a moderate positive correlation between students’ nursing professional values and spiritual intelligence scores. At the same time, it was found that nursing students’ compliance with professional values had a moderate positive correlation with self-understanding and a weak positive correlation with compassion and conscience (Table 3).

**Table 3:** Correlation of the spiritual intelligence scale and nurses professional values scale—revised

Scale	Nurses professional values scale—revised	
	*r*	*p*
Spiritual intelligence scale total score	.356**	**.000**
Self understanding	.344.**	**.000**
Human values	− .016	.777
Compassion	.224**	**.000**
Conscience	.141*	**.013**

Statistically signifcant values (*p* < .05) are shown in bold

*Correlation is significant at the 0.05 level (2-tailed)

**Correlation is significant at the 0.01 level (2-tailed)

Regression analysis revealed that professional values have a significant relationship with spiritual intelligence. Accordingly, spiritual intelligence explained 10% of the total variance in compliance with professional values (*R* = 0.328, *R*^2^ = 0.107, *p* < 0.001). As seen in Model 2, spiritual intelligence together with gender, choosing the profession willingly and having received spiritual training explained 12% of the total variance in compliance with professional values (*R* = 0.353, *R*^2^ = 0.125, *p* < 0.001). According to the model, spiritual intelligence was effective in adaptation to professional values, but gender, willingness to choose the profession and having received training on spirituality did not have a significant effect (Table 4).

**Table 4:** The predictive effect of spiritual intelligence on professional values

Model 1	B	SE	β	t	*p*	95.0% confidence interval	
						Lower	Upper
Constant	38.918	9.893		3.934	.000	19.453	58.383
Spiritual intelligence	.921	.151	.328	6.100	.000	.624	1.219
*R* = *0.328, R*^*2*^ = *0.107, F* = *37.216, p* < *0.001*							

Model 2	B	SE	β	t	*p*	95.0% confidence interval	
						Lower	Upper
Constant	39.042	10.175		3.837	***p* < *0.001***	19.020	59.065
Spiritual intelligence	.853	.154	.304	5.551	***p* < *0.001***	.551	1.156
Gender	.653	3.254	.011	.201	.841	− 5.751	7.057
Choosing the profession willingly	3.655	2.352	.086	1.554	.121	− .973	8.283
Receive training on spirituality	4.347	2.332	.100	1.864	.063	− .243	8.936
*R* = *0.353, R*^*2*^ = *0.125, F* = *10.910, p* < *0.001*							

Statistically signifcant values (*p* < .05) are shown in bold

(Gender coded as boy = 0, girl = 1: choosing the profession willingly yes = 1, no = 0: receive training on spirituality yes = 1, no = 0)

^a^Dependent variable: nurses professional values scale-revised

^b^Predictors: (constant), spiritual intelligence scale, gender, choosing the profession willingly, receive training on spirituality

## Discussion

In this study, which was conducted to determine the relationship between spiritual intelligence and compliance with professional values in nursing students, it was found that nursing students had high professional values. In an analytical cross-sectional study conducted by Yağcan et al. (2021) to determine the relationship between emotional intelligence and professional values in nursing students, it was reported that the mean total score of the professional values was 107.09 ± 15.70, and the mean scores of caring, professionalism and trust factors were 59.08 ± 9.02, 31.96 ± 5.30 and 11.92 ± 2.00, respectively. In another study evaluating the relationship between perception of professionalism and commitment to professional values in nursing students, the median score of the professional values scale of nursing students was 99 (Min = 47-Max = 130) (Peksoy et al., 2020). These results are similar to the findings of the current study. Professional values are action guidelines agreed upon by professional groups and experts and are of great importance as they provide a framework for the assessment of values and beliefs that influence professional performance (Bijani et al., 2019).

Spirituality is a need that is innate in the individual, has a different meaning for each individual and directs the feelings and thoughts of the individual in difficult situations such as illness and death (Arslan & Avcı, 2024; Erişen & Karaca Sivrikaya, 2017; Timmins & Calderia, 2017). George et al. (2022) found a significant weak positive correlation between spirituality and spiritual care perceptions of nursing students. In addition, it was stated in the study that spirituality is an important concept for the nursing discipline, that it creates meaningful results for the care of patients, and it was emphasized that it is an important aspect of holistic care (George et al., 2022). Spiritual intelligence is vital for nurses and nursing students to provide holistic and quality patient care, but not all nurses and nursing students have spiritual intelligence (Ahmadi et al., 2021). In the current study, the spiritual intelligence level of nursing students was found to be high in accordance with the literature. In a study conducted by Ahmadi et al. (2021) to determine the perceived professional competence in spiritual care and the predictive role of spiritual intelligence in nursing students, the mean score of students’ spiritual intelligence level was 67.86 ± 10.98. In another study conducted by Aliabadi et al. (2021) in three different hospitals in Iran, the spiritual intelligence levels of nurses were found between 64 and 65.50.

Spiritual intelligence refers to a deep understanding of existential questions and insight into different dimensions of life. With this skill, the nurse develops the ability to give more holistic care to the individual by increasing their inner awareness. With developing skills, the nurse acts in line with professional values. It is an indispensable part of nursing care that nurses, whose main responsibility is to provide care to individuals, act in compliance with their professional values. This situation is also very important for nursing students who will practice the nursing profession in the future (Beni et al., 2019; Sharifnia et al., 2022; Skrzypinska, 2021). In the current study, a significant and positive correlation was found between nurses’ spiritual intelligence levels and their compliance with professional values. It is also stated in the literature that as the level of spiritual intelligence increases, professional competence in spiritual care increases (Ahmadi et al., 2021). In addition, in this study, spiritual intelligence explained 10% of the total variance in compliance with professional values (*R* = 0.328, *R*^2^ = 0.107, *p* < 0.001). In the study conducted by Imani et al. (2021) to determine the relationship between spiritual intelligence and clinical competence, a significant relationship was found between spiritual intelligence and clinical competence (Imani et al., 2021).

In this study, self-understanding, compassion and conscience, which are sub-dimensions of spiritual intelligence, were found to be positively correlated with professional values. In the study conducted by Dincer and Çiftçi (2024) to examine the relationship between nursing students’ compassion competencies and their perceptions of spirituality and spiritual care, it was found that there was a moderate positive relationship between compassion competency and spirituality and spiritual care in nursing students. In addition, the students who participated in the study were found to have high levels of compassion competence and sensitivity to the needs of patients. Therefore, it has been reported that developing sensitivity, effective communication skills and insights, which are important dimensions of compassion competence, can improve the quality of nursing care (Dincer & Çiftçi, 2024). Considering that these parameters are in close relationship with professional values in nursing, it is seen that the results of the study positively affect professional values in nursing and agree with the results of the current study. In a cross-sectional study conducted by Severino-Gonzalez et al. (2022) on university students, the relationship between social responsibility and spiritual intelligence was examined. A significant correlation was found between university students’ social responsibilities and spiritual intelligence (Severino-Gonzalez et al., 2022). When the relationship between spiritual intelligence and communication skills of nursing students was examined, it was reported that there was a significant positive relationship between spiritual intelligence and communication skills (Shami et al., 2017). All these results support the positive relationship between spiritual intelligence and professional values found in the current study.

In this study, spiritual intelligence together with gender, choosing the profession willingly and having received spiritual education explained 12% of the total variance in compliance with professional values. However, according to the model, it was determined that spiritual intelligence was effective in compliance with professional values, but gender, willingness to choose the profession and having received training on spirituality did not have a significant effect (Table 4). When the literature is examined, it is concluded that nurses’ compliance with professional values does not vary according to gender, which supports our results (Avcı et al., 2019; Green, 2020). It is seen that there are different research results in the literature regarding the relationship between choosing the profession willingly and compliance with professional values. In the study conducted by Dündar et al. (2019), nurses’ desire for the profession, working willingly in the clinic and satisfaction with the profession had no effect on professional values. Anieche et al. (2022) found that career choice did not have a significant effect on professional values, but necessary guidance should be provided in career choice to protect professional values and images. In another study conducted by Meşe and Söylemez (2024) to determine the effect of nursing students’ choice of profession on professional behavior, a significant positive relationship was found between nursing students’ choice of profession and professional behavior. It can be said that the difference in the study results is due to the difference in the samples. When the results of Bekar and Arıkan’s study (2022) are examined, it is seen that there is no significant difference between nursing students’ knowledge about spirituality-spiritual care and professional values (Bekar & Arıkan, 2022). The current study is in parallel with the result that nursing students’ training on spirituality does not significantly affect professional values in nursing.

## Limitations

A limitation of the study is that it was conducted with nursing students from a single university in a single country and did not include the views of students from different cultures.

## Conclusions

As a result of the study, nursing students’ spiritual intelligence levels and their compliance with professional values were positively correlated. The higher the spiritual intelligence, the higher the compliance with professional values. The fact that professional values, which play an important role in holistic patient care, are related to spiritual intelligence shows that the development of spiritual intelligence should be given importance. In line with these results, it is recommended to raise awareness about spiritual intelligence in students and to take initiatives such as mindfulness practices, self-awareness programs, mentoring and counseling to increase students’ spiritual intelligence levels.
